# Nitrogen Gas Plasma Generated by a Static Induction Thyristor as a Pulsed Power Supply Inactivates Adenovirus

**DOI:** 10.1371/journal.pone.0157922

**Published:** 2016-06-20

**Authors:** Akikazu Sakudo, Yoichi Toyokawa, Yuichiro Imanishi

**Affiliations:** 1 Laboratory of Biometabolic Chemistry, School of Health Sciences, University of the Ryukyus, Nishihara, Okinawa, Japan; 2 Department of Virology, Research Institute for Microbial Diseases, Osaka University, Yamadaoka, Suita, Osaka, Japan; 3 NGK Insulators Ltd., Mizuho-ku, Nagoya, Japan; University of Nantes, FRANCE

## Abstract

Adenovirus is one of the most important causative agents of iatrogenic infections derived from contaminated medical devices or finger contact. In this study, we investigated whether nitrogen gas plasma, generated by applying a short high-voltage pulse to nitrogen using a static induction thyristor power supply (1.5 kilo pulse per second), exhibited a virucidal effect against adenoviruses. Viral titer was reduced by one log within 0.94 min. Results from detection of viral capsid proteins, hexon and penton, by Western blotting and immunochromatography were unaffected by the plasma treatment. In contrast, analysis using the polymerase chain reaction suggested that plasma treatment damages the viral genomic DNA. Reactive chemical products (hydrogen peroxide, nitrate, and nitrite), ultraviolet light (UV-A) and slight temperature elevations were observed during the operation of the gas plasma device. Viral titer *versus* intensity of each potential virucidal factor were used to identify the primary mechanism of disinfection of adenovirus. Although exposure to equivalent levels of UV-A or heat treatment did not inactivate adenovirus, treatment with a relatively low concentration of hydrogen peroxide efficiently inactivated the virus. Our results suggest the nitrogen gas plasma generates reactive chemical products that inactivate adenovirus by damaging the viral genomic DNA.

## Introduction

Adenoviruses commonly cause infection of the respiratory system as well as a variety of other illnesses, including pharyngoconjunctival fever (pool fever) and epidemic keratoconjunctivitis, depending on the particular viral serotype [1]. The adenovirus particles can be transmitted by direct contact, fecal-oral transmission, or waterborne transmission. Human adenovirus is an important causative agent of iatrogenic infections resulting from contaminated medical devices and finger contact, especially in ophthalmology. Additionally, type 40 and 41 adenoviruses, which are often spread by fecal-oral transmission, are important causative agents of acute gastroenteritis in children. A common cause of iatrogenic infection is from the use of medical instruments, especially ophthalmic medical devices, which have not been properly sterilised. Adenovirus is resistant to 70% isopropyl alcohol, which is often used to swab down medical equipment [2]. A common way of sterilsing medical devices is autoclaving (121°C, 20 min) or treatment with a 1:10 dilution of household bleach containing about 6,000 ppm chlorine. However, medical instruments are sometimes sensitive to treatment with heat (e.g., rubber components) or bleach (e.g., rubber or metal components). As an alternative, several germicides can be used to eliminate adenovirus, such as treatment with peracetic acid or aldehyde (glutaraldehyde and *ortho*-phthalaldehyde) [2]. However, the use of these compounds as disinfectants is restricted because of their toxicity. Although treatment with 65–70% ethanol is reasonably efficient for the disinfection of adenovirus, the presence of organic matter (i.e., fetal calf serum) renders such treatment ineffective [2]. Consequently, a low temperature, non-chemical method is required to efficiently disinfect medical instruments, especially ophthalmic devices. Gas plasma devices can generate chemical species with high reactivity at low temperatures. Thus, a sterilization method using gas plasma should be ideal for disinfecting heat-sensitive medical devices.

We recently developed a nitrogen gas plasma instrument (BLP-TES) that delivers a short high-voltage pulse to nitrogen gas *via* a static induction (SI) thyristor power supply to generate a gas plasma [3, 4]. Moreover, we have shown that this nitrogen gas plasma can efficiently inactivate not only bacteria, but also bacterial spores and endotoxins [3, 5]. In addition, we have reported biochemical changes and inactivation of influenza virus after nitrogen gas plasma treatment. Based on the promising results from these previous investigations, we aimed to examine the potential use of nitrogen gas plasma for the inactivation of adenovirus.

Adenoviruses are medium sized viruses, composed of a complex protein capsid surrounding a double-stranded DNA genome (approximately 36 kbp) and core proteins [6]. The core proteins bind to the viral genomic DNA and form a core within the internal region of the viral particle. The capsid itself is composed of hexon and penton proteins, where fiber proteins extend, as well as other proteins (IIIa, VI, VIII, IX). Here, potential changes to the viral DNA and proteins after nitrogen gas plasma treatment were investigated by biochemical analyses. Previous studies have shown that the nitrogen gas plasma device (BLP-TES) generates heat, ultraviolet (UV-A) radiation and reactive chemical products (hydrogen peroxide-like species) [7, 8]. In order to clarify the mechanism(s) by which nitrogen gas plasma inactivates adenovirus, we subjected the virus to each of these factors independently. The relative contribution of each disinfection factor was then estimated.

## Materials and Methods

### Adenovirus and cells

Genetic recombinants of adenovirus type 5 (all with a titer of 1.0 x 10^9^ plaque-forming units per ml (PFU/ml)), which lack the ability to grow by deletion of essential viral gene E1, including Axcw2, AxCAwt2 and AxCAiLacZ, were purchased from Takara Bio Inc. (Otsu, Japan). The virus preparation methods are available from the company’s literature (Adenovirus Dual Expression kit) [9] and US patent [10]. This defective virus is able to infect human as well as non-human cells, but cannot replicate. Nonetheless, proliferation of the defective virus is made possible by using a packaging cell line, such as HEK293 cells, which express the E1 gene of adenovirus. Specifically, the E1 gene is integrated into the genome of HEK293 cells, enabling them to support replication of E1-deleted as well as wild type virus [11, 12]. As HEK293 cells allow completion of the viral productive cycle, they are used for determination of viral titers and other infection assays (5-bromo-3-indolyl-β-D-galactopyranoside assay and infectious titer assay using an antibody against adenovirus hexon protein). HEK293 cells were cultured in DMEM (high glucose, 4.5g/L D-(+) glucose) (Nacalai Tesque, Kyoto, Japan) supplemented with 10% fetal bovine serum at 37°C in a 5% CO_2_ atmosphere. Axcw2 is adenovirus type 5 alone. AxCAwt2 contains adenovirus type 5, CAG promoter (cytomegalovirus enhancer + chicken β-actin promoter), and rabbit β-globin polyA site. AxCAiLacZ contains adenovirus type 5, CAG promoter (cytomegalovirus enhancer + chicken β-actin promoter), rabbit β-globin polyA site, and *Escherichia coli* β-galactosidase gene (Takara Bio Inc).

### Gas plasma

BLP-TES (NGK Insulators Ltd., Nagoya, Japan) was used as a nitrogen gas plasma device, which employs a short high-voltage pulse *via* a SI thyristor power supply [4]. A cathode electrode (earth electrode) was placed between the anode electrodes (high voltage electrodes). A 20 μL aliquot of cell culture medium containing adenovirus spotted onto glass coverslips and indicator strips were placed on the grid of the earth electrode. The operation procedure for nitrogen gas plasma production was as follows. Firstly, the chamber box containing the samples was decompressed and degassed, and then nitrogen gas (99.9995%; Okano, Okinawa, Japan) was introduced. The pressure in the box was maintained at about 0.5 atmospheres during the electrical discharge at 1.5 kpps (kilo pulse per second). The nitrogen gas plasma treated and untreated adenovirus samples on the glass coverslips were then recovered by resuspension in phosphate buffered saline (PBS) for analysis.

### Viral titration assay by calculating plaque forming units (PFUs)

PFUs per ml were determined by performing 2-fold serial dilutions of samples in 96-well plates containing 6 x 10^4^ cells/well of HEK293 cells (ATCC CRL1573^TM^) as described by the manufacturer (Adeno-X^TM^ Expression System User Manual, Clontech Laboratories Inc.) [13]. Cells were washed with PBS prior to infection. Infected cells were incubated for 5 days at 37˚C in a 5% CO_2_ atmosphere. To determine the virus titer, plaques caused by the cytopathic effect of viral proliferation were counted. To minimize experimental error, only plates containing between 10 and 100 plaques were counted, depending on the size of the cell culture plate used. According to statistical analysis, when 100 plaques are counted the sample titer will vary by plus or minus 10%.

### 5-bromo-3-indolyl-β-D-galactopyranoside staining

AxCAiLacZ vector encodes recombinant human adenovirus type 5 carrying the lacZ gene as a reporter. Production of β-galactosidase can be readily monitored using 5-bromo-3-indolyl-β-D-galactopyranoside, which is an alternative β-galactosidase chromogenic substrate to 5-bromo-4-chloro-3-indolyl-β-D-galactopyranoside (X-gal), with the X-Gal Staining^TM^ kit (Genlantis, San Diego, CA). Briefly, cells were fixed with formaldehyde-glutaraldehyde buffer and washed with PBS. Staining solution of 5-bromo-3-indolyl-β-D-galactopyranoside was added and the samples were incubated for 18 h at 37°C in a CO_2_ incubator. Blue cells, indicating infection with adenovirus, were subsequently visualized by optical microscopy.

### Infectious titer assay using an antibody against adenovirus hexon protein

The Adeno-X^TM^ rapid titer kit (Clontech Laboratories Inc., Mountain View, CA) is infectious titer assay based on detection of adenovirus hexon protein expression by immunostaining of infected cells. Briefly, viral samples were added to HEK293 cells and incubated for 48 h. Samples were then fixed with ice-cold 100% methanol and the cells stained with mouse anti-adenovirus hexon antibody. The signal was detected with a rat anti-mouse antibody conjugated to horseradish peroxidase and developed with metal-enhanced 3,3’-diaminobenzidine tetrahydrochloride (DAB). Brown/black cells, indicating infection with adenovirus, were subsequently visualized by optical microscopy.

### Viral DNA extraction and DNA-polymerase chain reaction (PCR)

Adenovirus DNA in the recovered solution from dried spots on coverslips before and after nitrogen gas plasma treatment were extracted using the QIAamp Viral RNA mini kit (Qiagen, Hilden, Germany). Reproducible extraction of adenovirus DNA by this method has been confirmed before and after capturing and concentrating adenovirus using magnetic anionic nanobeads [14]. Plasma-treated and untreated samples (20 μL) were solubilized in lysis buffer (560 μL Buffer AVL containing carrier RNA). The extracted DNA was bound to a column and then eluted in 60 μl of nuclease-free water. Viral DNA was amplified in a reaction mixture of 20 μL containing 2 μL of the eluted viral DNA as well as primers (0.2 μL each, 100 pmol/μL), Ex Taq (Takara Bio Inc.) (0.1 μL, 5 units/μL), and 10x Ex Taq buffer (2 μL), MgCl_2_ (1.6 μL, 25 mM), dNTP mixture (1.6 μL, 2.5 mM each) and water (12.3 μL) under the following conditions: 30 cycles of 94°C for 30 sec, 50°C for 30 sec, and 72°C for 2 min. PCR was carried out using the following primers specific for the adenovirus hexon gene;

Hexon-F: 5'-TGGGTGATAACCGTGTGCTA-3 ',

Hexon-R: 5'-TTAATGCTAGCCCCGTCAAC-3'

The PCR products were analysed by agarose gel electrophoresis on a 1.2% gel.

### Real-time PCR

Extracted viral DNA as described in the above section was also analysed by real-time PCR using SYBR Premix Ex TaqII (Tli RNase H plus) (Takara Bio Inc.) according to the manufacturer’s instructions. Briefly, the real-time PCR mixture of 25 μL included 2 μL of the extracted viral DNA, 2x SYBR Premix Ex Taq II (12.5 μL), and the forward and reverse target gene primers (1 μL each, 10 μM): realAdenoHexon-F, 5’-GACATGACTTTCGAGGTCGATCCCATGGA-3’; realAdenoHexon-R, 5’-CCGGCTGAGAAGGGTGTGCGCAGGTA-3’ and water (8.5 μL). A Thermal Cycler Dice Real Time System (Takara Bio Inc.) was used to amplify the DNA. Reactions were performed by denaturation at 95°C for 30 sec followed by 40 cycles of 95°C for 30 sec and 60°C for 30 sec. Each reaction was done in quadruplicate. Reactions were analyzed using the Thermal Cycler Dice Realtime System Single software (Takara Bio Inc.). The relative DNA levels of each sample were compared with serially diluted viral DNA and estimated using the standard curve of diluted viral DNA versus absorbance. PCR specificity was verified by dissociation curve analysis of the amplified DNA fragments of step 1 (95°C/15 sec), step 2 (60°C/30 sec), and step 3 (95°C/15 sec).

### DNA sequencing

The products of conventional PCR and real-time PCR were purified and cloned into pT7Blue T-vector (Novagen, Madison, WI). Each amplified product was verified by DNA sequencing using an ABI PRISM3100 Genetic Analyzer (Applied Biosystems, Foster City, CA). R-20mer and U-19mer primer (Novagen) were used in the sequencing reactions.

### Immunochromatography

Adenovirus antigens were detected using the Quick Navi-Adeno kit (Denka Seiken Co., Ltd., Tokyo, Japan) according to the manufacturer’s instructions. This immunochromatography kit specifically recognizes adenovirus hexon protein [15]. Band intensity of the test line was analysed by densitometric analysis with ImageJ software (National Institutes of Health, Bethesda, Maryland, USA).

### Western blotting

Each fraction was solubilized in an equal volume of 2× sodium dodecyl sulfate (SDS) gel-loading buffer [90 mM Tris–HCl (pH 6.8), 10% mercaptoethanol, 2% SDS, 0.02% bromophenol blue, and 20% glycerol] and boiled for 5 min. Proteins were then resolved by SDS–polyacrylamide gel electrophoresis (PAGE) using an 8% gel before being electroblotted onto a polyvinylidene difluoride (PVDF) membrane (Hybond-P; Amersham-Pharmacia Biotech, Piscataway, NJ) for 60 min at 15 V. Blots were treated with 5% skimmed milk for 1 h at room temperature and then incubated with a goat anti-adenovirus polyclonal antibody (AP00664PU-N, Acris) in PBS containing 0.1% Tween 20 (PBS-T) and 0.5% skimmed milk for 1 h at room temperature. After three washes with PBS-T, the membrane was incubated in horseradish peroxidase (HRP)-conjugated anti-goat IgG (Jackson ImmunoResearch Laboratories, Inc., West Grove, PA) in PBS-T and 0.5% skimmed milk for 1 h at room temperature. After three washes with PBS-T, the probed proteins were detected using an enhanced chemiluminescence detection kit (Amersham-Pharmacia Biotech). The signal of chemiluminescence was detected by using Ez-Capture MG (ATTO Corp., Tokyo, Japan). Band intensity was analysed by densitometric analysis with ImageJ software (National Institutes of Health).

### Measurement of reactive chemical products

An appropriate indicator paper was used to determine the concentration of various reactive chemical species produced during exposure to nitrogen gas plasma. Specifically, Merckoquant^®^ Peroxide Test 0.5–25 mg/l H_2_O_2_, Merck KGaA, Darmstadt, Germany), Merckoquant^®^ Nitrate Test 10–500 mg/l NO_3_^-^ (Merck KGaA), and (Merckoquant^®^ Nitrite Test 2–80 mg/l NO_2_^-^ (Merck KGaA) were used to monitor the generation of hydrogen peroxide, nitrate (NO_3_^-^) and nitrite (NO_2_^-^), respectively. The chemical indicator was placed on the earth electrode and exposed to nitrogen gas plasma in order to measure the concentration of the various chemical species during operation of the nitrogen gas plasma instrument (BLP-TES). After nitrogen gas plasma plasma treatment, the strip was immediately dipped in distilled water and then scanned to generate an image. The change in the colour of the strip was converted to an RGB code and compared with a standard curve developed from the RGB code of a reference strip.

### Statistical analysis and repetition

The results are the mean ± standard deviation of replicate experiments (*N* = 3–4). All results were confirmed by at least two independent analyses. The statistical analysis of significant difference was performed by the non-repeated measures ANOVA followed with the Bonferroni correction.

## Results

**Fig 1:**
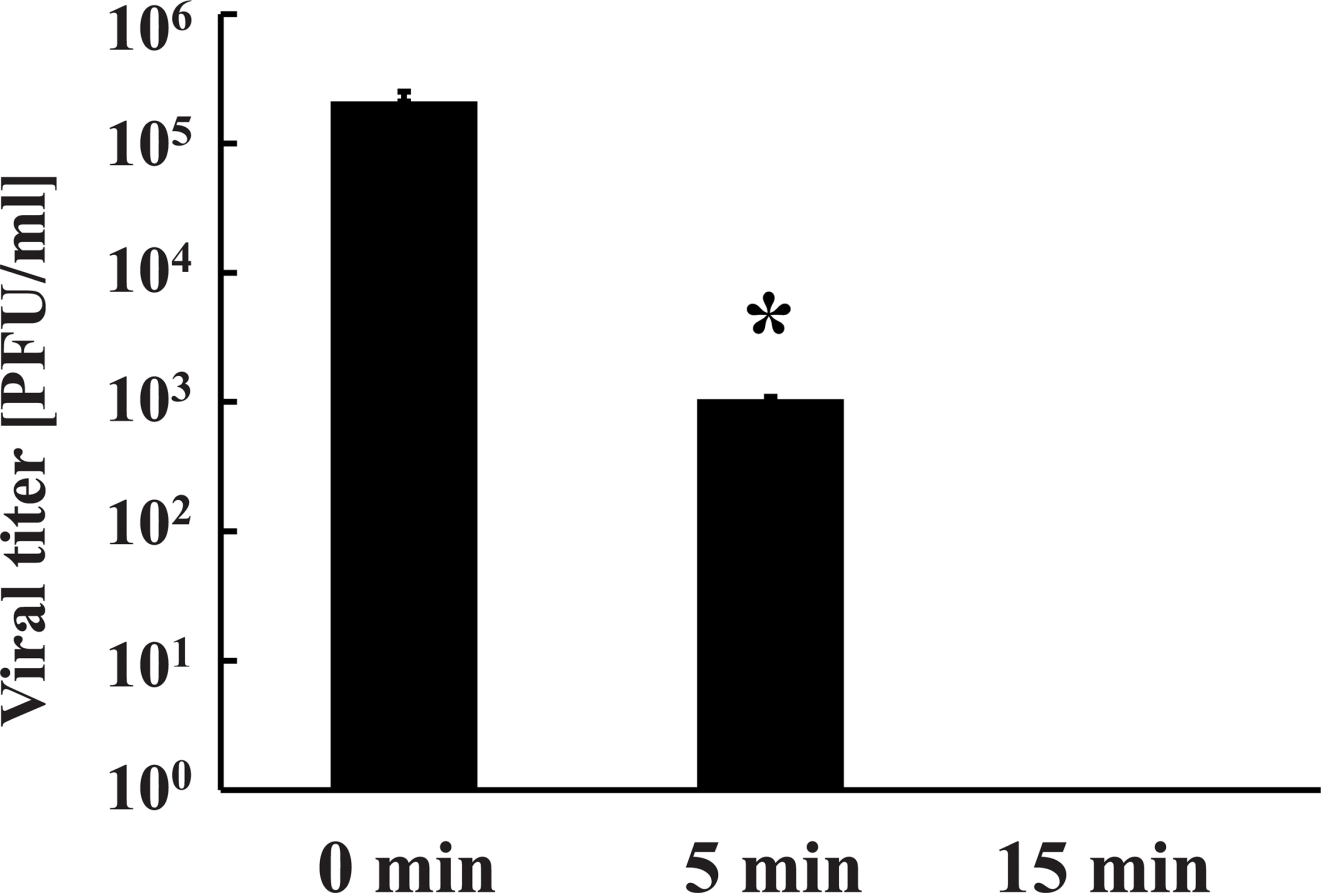
Viral titration assays demonstrate that nitrogen gas plasma treatment of adenovirus results in a decrease in viral titer. A cell culture medium of adenovirus vector (AxCAwt2)-infected HEK293 cells (1.0 x 10^9^ PFU/ml) was air-dried onto a glass coverslip and treated with nitrogen gas plasma using a BLP-TES device (NGK Insulators, Ltd., Nagoya, Japan) at 0.5 atmospheric pressure. The recovered samples were then subjected to a viral titration assay using HEK293 cells as described in Materials and Methods. The number of plaque forming units (PFU) per ml of adenovirus treated with nitrogen gas plasma at 1.5 kpps for 0, 5, or 15 min (*N* = 4) was then determined. The 0 min samples are recovered from coverslips subjected to 0.5 atmospheric pressure without plasma treatment. Differences where *p*<0.05(*) versus control (0 min) were considered significant. No plaques were detected after treatment for 15 min.

**Fig 2:**
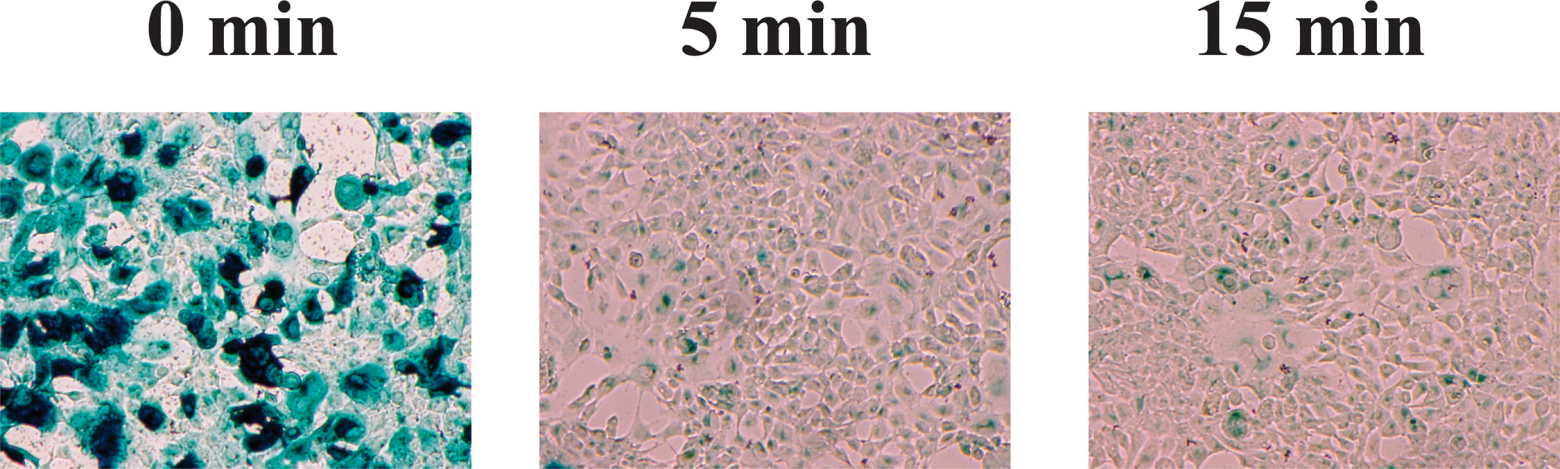
5-bromo-3-indolyl-β-D-galactopyranoside staining detected a decrease in viral titer of adenovirus after nitrogen gas plasma treatment. A cell culture medium of adenovirus vector (AxCAiLacZ)-infected HEK293 cells (1.0 x 10^9^ PFU/ml) was air-dried onto a glass coverslip and treated with nitrogen gas plasma using the BLP-TES device at 1.5 kpps for 0, 5, and 15 min. After the treatment, adenovirus was recovered from the spots and subjected to 5-bromo-3-indolyl-β-D-galactopyranoside staining as described in Materials and Methods.

**Fig 3:**
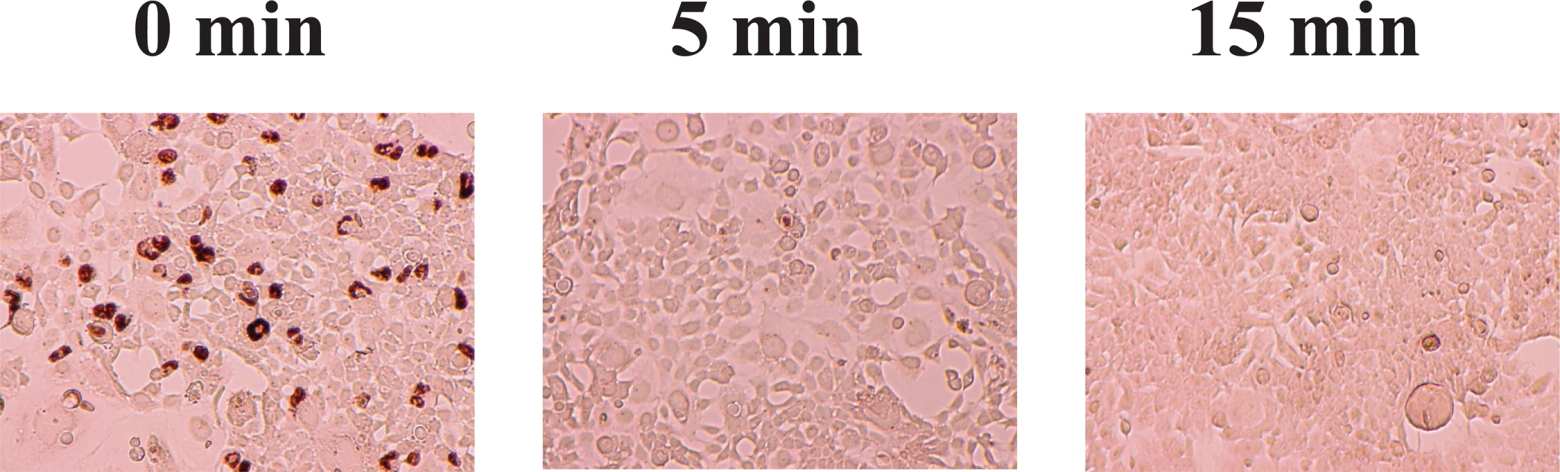
Infectious titer assay using antibody against adenovirus hexon protein show decreased viral infectivity of adenovirus after nitrogen gas plasma treatment. A cell culture medium of adenovirus vector (AxCAwt2)-infected HEK293 cells (1.0 x 10^9^ PFU/ml) was air-dried onto a glass coverslip and treated with nitrogen gas plasma using a BLP-TES device at 1.5 kpps for 0, 5, and 15 min. After treatment, adenovirus was recovered from the spots and then subjected to an infectious titer assay using antibody against adenovirus hexon protein to determine the number of infected cells.

The aim of this study was to investigate the effect of nitrogen gas plasma on adenovirus. An aliquot of cell culture medium containing adenovirus vector (AxCAwt2)-infected HEK293 cells was spotted onto a glass coverslip (20 μl/coverslip), air-dried and then subjected to nitrogen gas plasma treatment (1.5 kpps) using a BLP-TES device. Following treatment, samples on the coverslip were recovered and analysed using a variety of techniques. Infectivity of the recovered sample from the coverslips was measured using a viral titration assay (Fig 1). The viral titer of samples at 0 min, recovered from coverslips subjected to 0.5 atmospheric pressure without plasma treatment, was 2.1 x 10^5^ PFU/ml, but significantly decreased to 1.0 x 10^3^ PFU/ml after treatment with nitrogen gas plasma for 5 min and no viable cell count was detected after 15 min treatment. Thus, adenovirus was inactivated to a level below the detectable limit of our assay within 15 min of exposure to the nitrogen gas plasma. Next, we examined the effect of nitrogen gas plasma treatment on the inactivation of adenovirus derived from AxCAiLacZ vector using either 5-bromo-3-indolyl-β-D-galactopyranoside staining for adenovirus infectivity or infectious titer assay using antibody to detect adenovirus hexon protein. In the 5-bromo-3-indolyl-β-D-galactopyranoside assay, production of β-galactosidase only occurs if the infectivity of the virus is not impaired and the viral DNA is undamaged. A large number of blue-stained cells, indicating virus infectivity and expression of β-galactosidase, were detected at 0 min after infection with virus samples recovered from coverslips subjected to 0.5 atmospheric pressure without plasma treatment. However, no stained cells were observed after infection with virus subjected to nitrogen gas plasma treatment for 5 or 15 min (Fig 2). Infectious titer assay using antibody against adenovirus hexon protein also detected the viral infectivity (i.e., brown stained-cells) in the 0 min sample, corresponding to virus samples recovered from coverslips subjected to 0.5 atmospheric pressure without plasma treatment, whereas no stained cells were observed for virus samples subjected to 5 or 15 min gas plasma treatment (Fig 3). Thus, the results from both 5-bromo-3-indolyl-β-D-galactopyranoside staining and the infectious titer assay using anti-adenovirus antibody concur with the findings from the viral titration assays. Taken together, these experiments suggested adenovirus is inactivated by treatment with nitrogen gas plasma.

**Fig 4:**
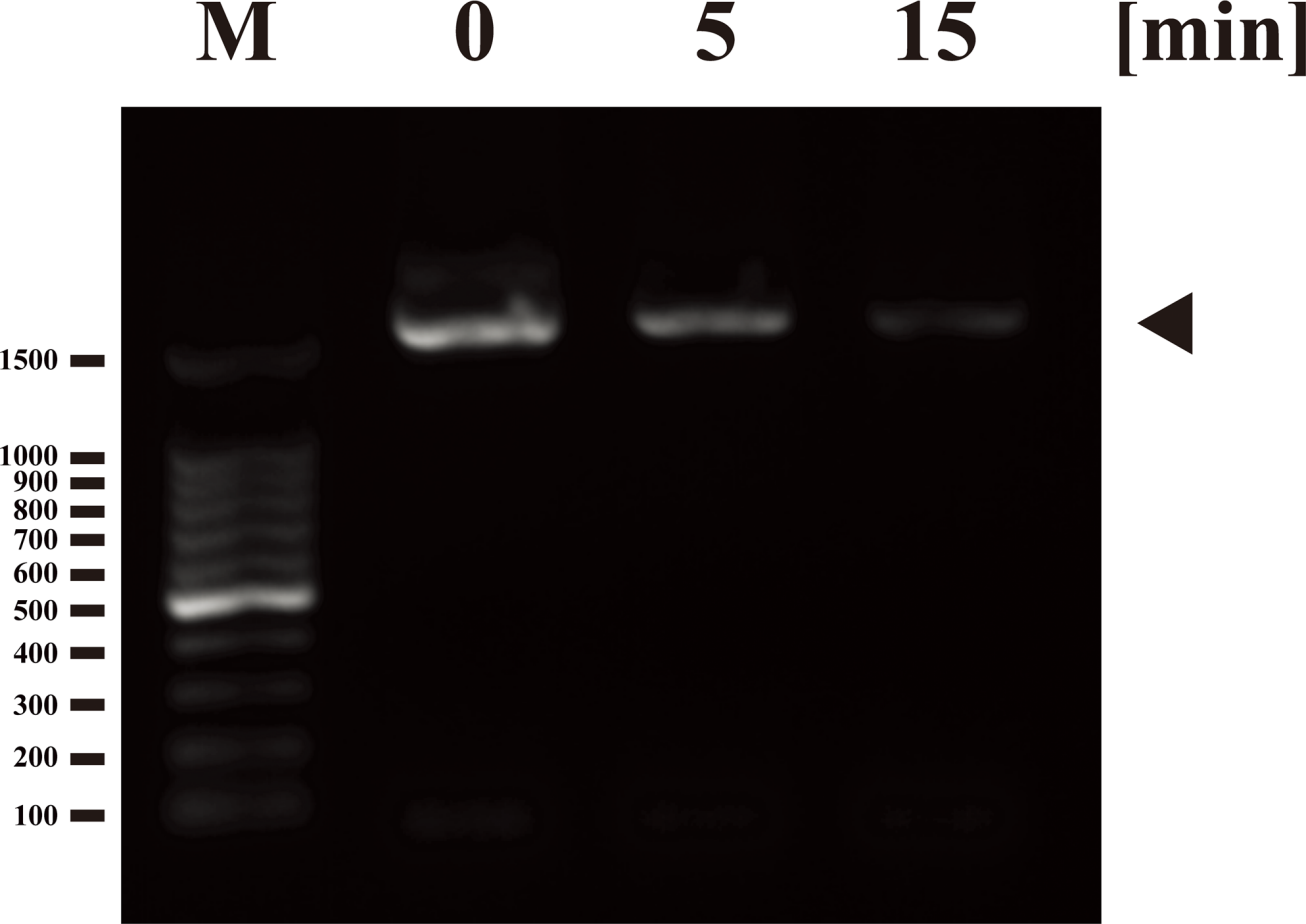
Nitrogen gas plasma treatment induces damage to the genomic DNA of adenovirus. A cell culture medium of adenovirus vector (AxCAiLacZ)-infected HEK293 cells (1.0 x 10^9^ PFU/ml) was air-dried onto a coverslip and treated with nitrogen gas plasma using the BLP-TES device at 1.5 kpps for 0, 5 and 15 min. The recovered samples were then subjected to polymerase chain reaction (PCR) analysis using primers designed to amplify the hexon gene of adenovirus. A PCR product of the anticipated size was observed at 0 min (indicated by arrowhead), but the intensity of the band progressively diminished after gas plasma treatment for 5 and 15 min. The molecular marker lane (M) is 100 kb DNA ladder. Numbers shown on the left correspond to the molecular size (bp).

**Fig 5:**
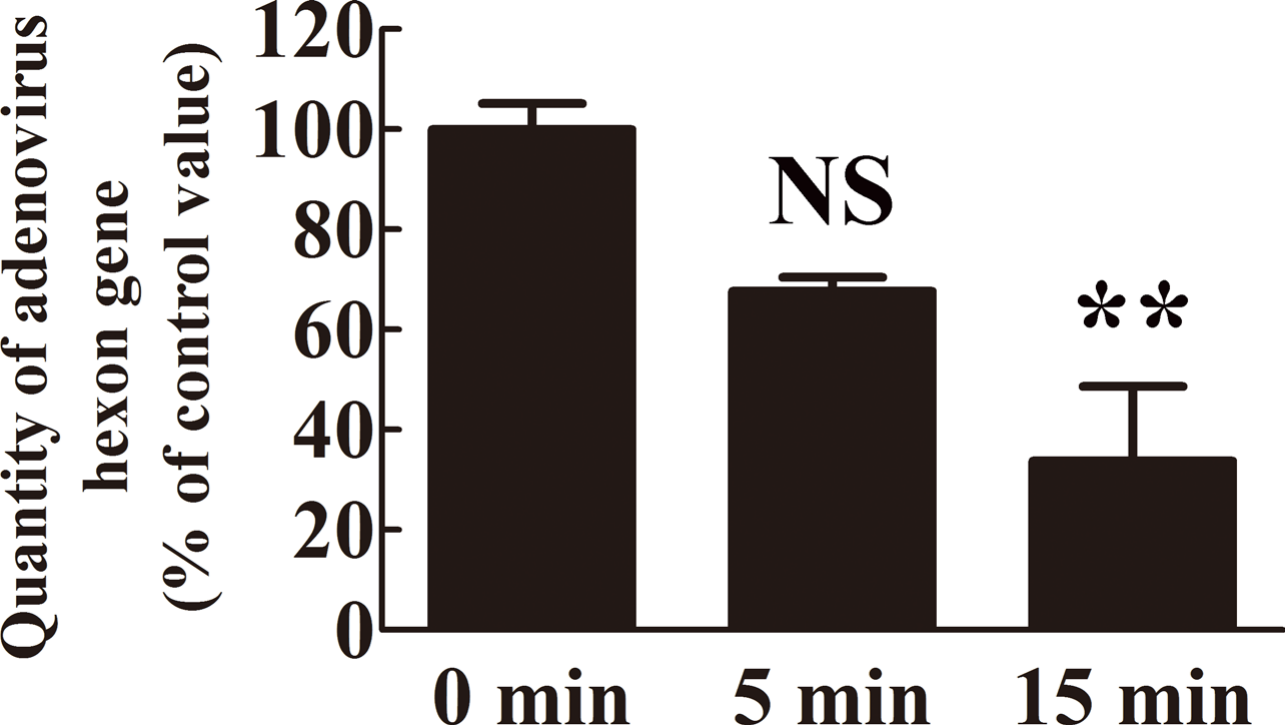
The level of intact viral genomic DNA decreased following nitrogen gas plasma treatment. A real-time PCR assay was performed using cell culture medium of HEK293 cells infected with AxCAiLacZ vector (1.0 x 10^9^ PFU/ml) after treatment with nitrogen gas plasma for 0, 5, and 15 min (*N* = 4). The primers were designed against the adenovirus hexon gene. The amount of intact adenovirus genomic DNA decreased after nitrogen gas plasma treatment. The average titer of 0 min samples was taken as 100%. Differences where *p*<0.01(**) versus control (0 min) were considered significant or not significant (NS).

Next, we investigated the effect of nitrogen gas plasma on the adenovirus genome. PCR analysis of adenovirus derived from AxCAiLacZ vector showed a decreased band intensity corresponding to the hexon gene in viral samples treated with nitrogen gas plasma for 5 and 15 min by comparison to the same DNA product amplified from the untreated control samples (0 min) (Fig 4). Because the results from DNA-PCR were only qualitative, we used real-time PCR for quantitative analysis. Thus, the amount of adenovirus hexon gene as an index of intact viral DNA was analyzed using real-time PCR (Fig 5), where the average quantity of DNA at 0 min was standardized as 100%. Compared to the 0 min sample (100±5.18%), the level of intact viral genomic DNA decreased to 67.69±2.75% after 5 min of nitrogen gas plasma treatment and 33.79±14.83% after 15 min of treatment. The sequence of the amplified product (1597-bp in length) corresponded to the adenovirus type 5 hexon gene (i.e., identity to Genbank accession number AC_000008: 94% using the forward primer and 98% using the reverse primer). In addition, the 140-bp band obtained by real-time PCR was also confirmed to correspond with part of the hexon gene of adenovirus type 5 (i.e., sequence identity to Genbank accession number AC_000008: 96% using the forward primer and 97% using the reverse primer). Taken together, these results suggested that the reduced level of PCR amplification in adenovirus samples treated with nitrogen gas plasma was due to viral DNA damage.

**Fig 6:**
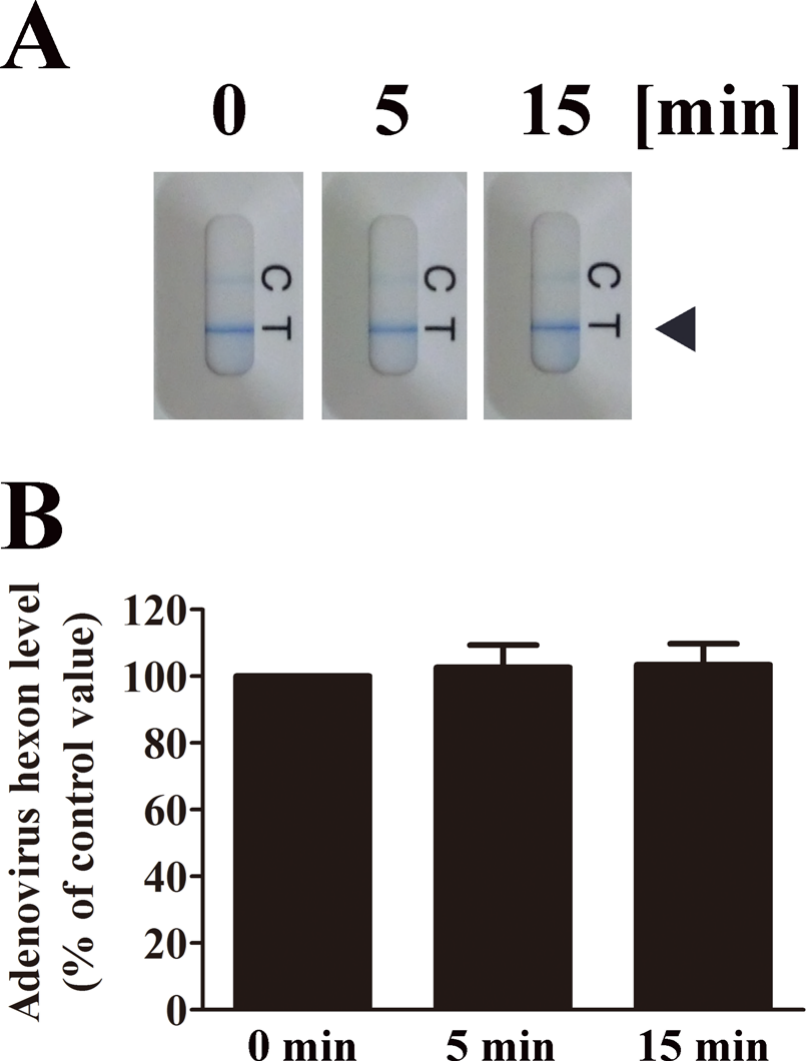
Nitrogen gas plasma treatment has no effect on adenovirus hexon protein. (A) Nitrogen gas plasma-treated cell culture medium of HEK293 cells infected with adenovirus vector (AxCAiLacZ) (1.0 x 10^9^ PFU/ml) was subjected to immunochromatography using the Quick Navi-Adeno kit (Denka Seiken Co., Ltd., Tokyo, Japan). The bands corresponding to adenovirus hexon protein are indicated by an arrowhead. C: Control line; T: Test line. (B) The band intensities of test lines of the immunochromatography in three independent experiments were compared using Image J software. The band intensity of the test line for the 0 min sample in each experiment was taken as 100%. Band intensities of test lines of adenovirus samples treated for 0, 5 and 15 min are shown. Immunochromatography showed no significant change in the hexon protein of adenovirus after nitrogen gas plasma treatment for up to 15 min.

Next, we investigated whether nitrogen gas plasma degrades viral capsid using either immunochromatography or Western blotting. Immunochromatography showed that the level of adenovirus hexon protein, which forms part of the viral capsid, is not degraded after nitrogen gas plasma treatment for 5 or 15 min compared to the untreated sample (0 min) (Fig 6). The band intensities of test lines obtained from three independent immunochromatography assays were then compared using ImageJ software. The band intensity of test lines corresponding to each 0 min sample of adenovirus was taken as 100%. The data indicates no significant difference between samples treated for 5 min (102.66±6.71) or 15 min (103.48±6.27) and untreated (0 min) samples.

**Fig 7:**
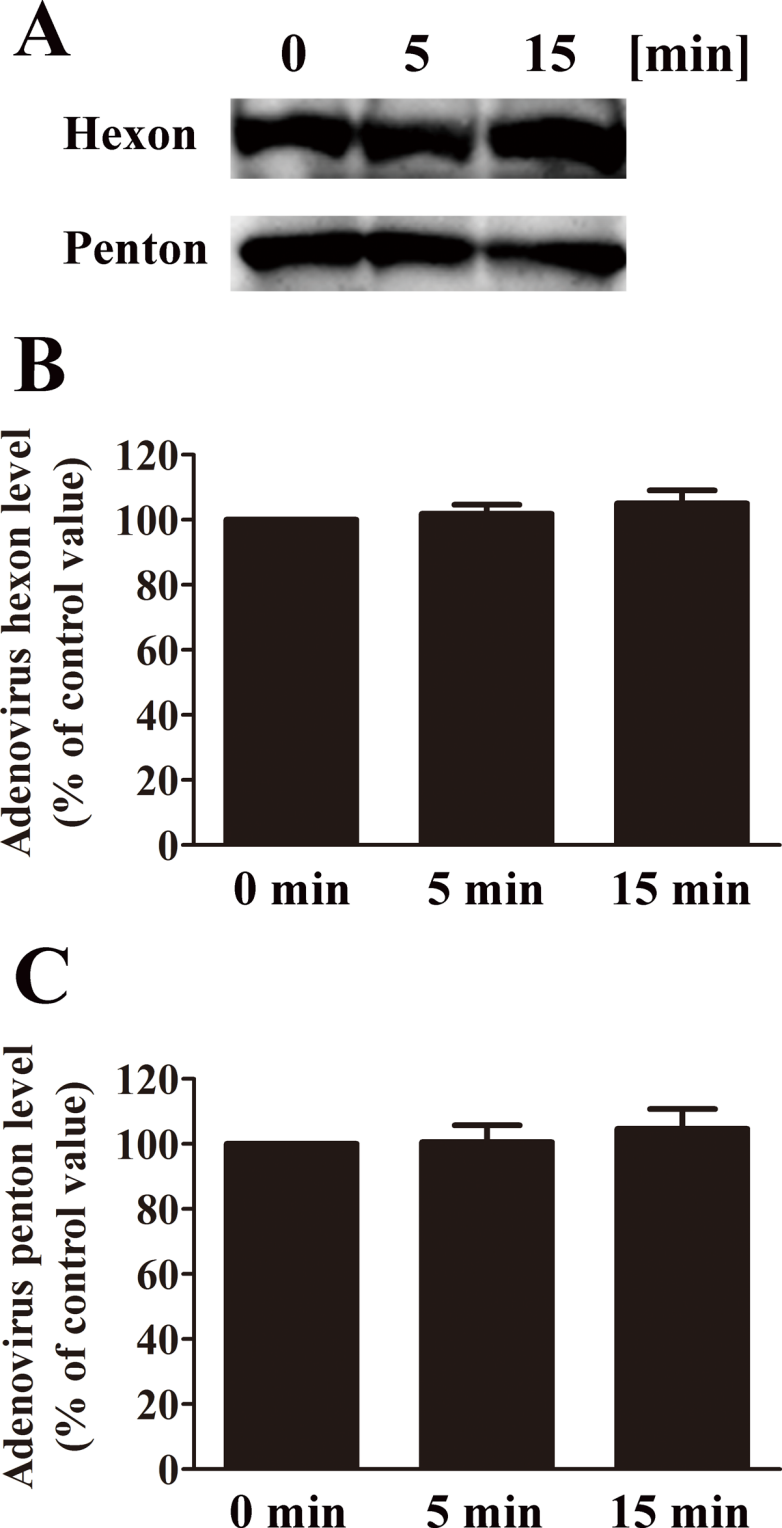
No difference in the adenovirus hexon and penton proteins was detected after nitrogen gas plasma treatment. (A) Cell culture medium of adenovirus vector (AxCAiLacZ)-infected HEK293 cells (1.0 x 10^9^ PFU/ml) was treated with nitrogen gas plasma for 0, 5 or 15 min (BLP-TES, 1.5 kpps). The treated adenovirus samples were then subjected to Western blotting with a polyclonal anti-adenovirus antibody. (B) The band intensities from three independent Western blots were compared using Image J software. The band intensity for 0 min in each blot was taken as 100%. Band intensities of adenovirus samples treated for 0, 5 and 15 min are shown. No significant change was detected in the hexon (116 kDa) and penton (80 kDa) capsid proteins of adenovirus after nitrogen gas plasma treatment for up to 15 min.

Western blotting was also performed to investigate the effect of nitrogen gas plasma on viral proteins derived from adenovirus vector AxCAiLacZ (Fig 7). These studies showed the intensity of the band corresponding to hexon protein (116 kDa) and penton protein (80 kDa) [16] were unchanged after nitrogen gas plasma treatment for 5 or 15 min compared to the untreated sample. The intensities of the bands corresponding to hexon and penton protein obtained from three independent Western blots were then compared using ImageJ software. The band intensity corresponding to each 0 min sample of adenovirus was taken as 100%. The intensity of the band corresponding to hexon protein indicates no significant difference between samples treated for 5 min (101.88±2.78) or 15 min (105.04±3.99) with untreated (0 min) samples. Similarly, the intensity of the band corresponding to penton protein displays no significant difference between samples treated for 5 min (100.59±5.21) or 15 min (104.66±6.11) with untreated (0 min) samples.

**Fig 8:**
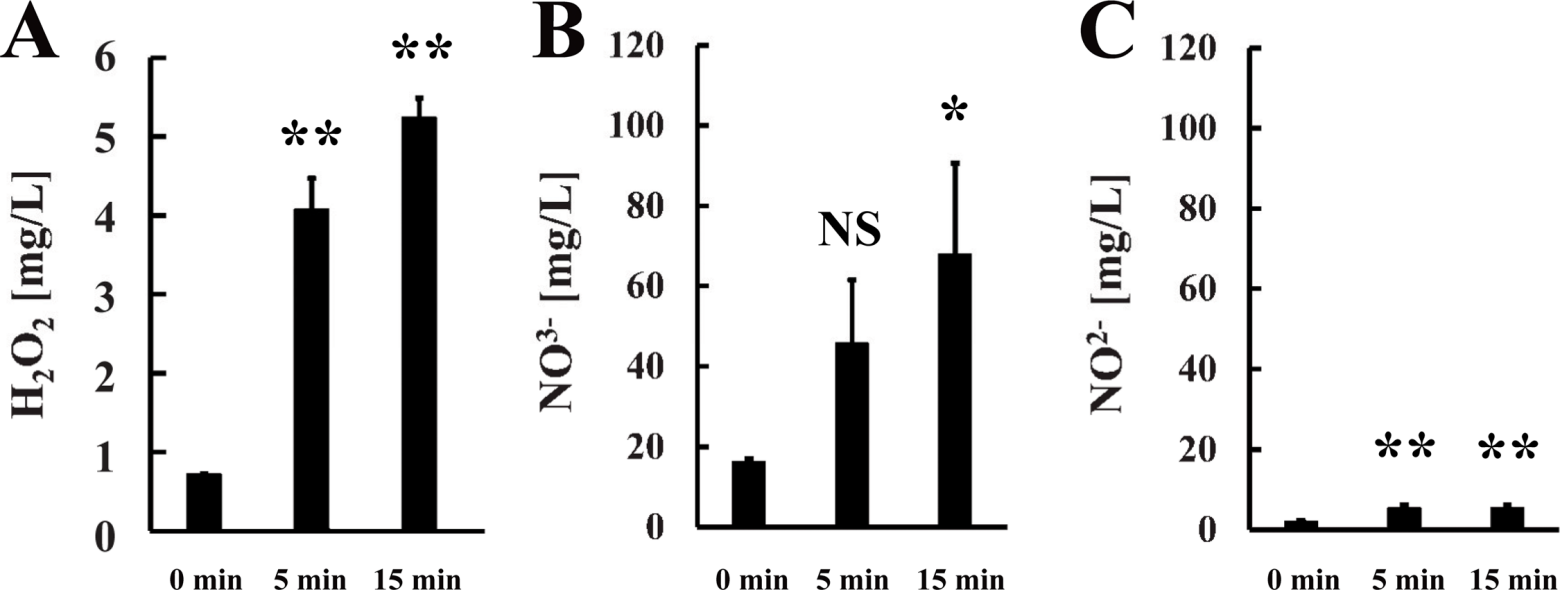
Reactive chemical species are generated during operation of the nitrogen gas plasma instrument. Chemical indicator strips were used to detect the generation of reactive chemical species during operation of the nitrogen gas plasma device. Specifically, we tested for hydrogen peroxide [Merckoquant^®^ Peroxide Test 0.5–25 mg/l H_2_O_2_, Merck KGaA, Darmstadt, Germany] (A), nitrate (NO_3_^-^) [Merckoquant^®^ Nitrate Test 10–500 mg/l NO_3_^-^, Merck KGaA] (B), and nitrite (NO_2_^-^) [Merckoquant^®^ Nitrite Test 2–80 mg/l NO_2_^-^, Merck KGaA] (C). In each case, the strip was placed on the earth electrode of the BLP-TES instrument. Nitrogen gas plasma treatment was then performed at 1.5 kpps for 0, 5, or 15 min (*N* = 3). After treatment, the strips were immediately dipped into pure water (Otsuka distilled water; Otsuka Pharmaceutical Co. Ltd., Tokyo, Japan). The concentration of hydrogen peroxide, nitrate, and nitrite was estimated from the change in colour of the corresponding chemical indicator strip, which was scanned and compared with a calibration curve of references. Differences where *p*<0.05(*) and *p*<0.01(**) versus control (0 min) were considered significant or not significant (NS).

Next, we estimated the levels of reactive chemical species generated during operation of the nitrogen gas plasma device (Fig 8). The hydrogen peroxide level depended on the length of the plasma treatment and was estimated to be 4.08±0.40 mg/L at 5 min and 5.24±0.24 mg/L at 15 min. The production of other reactive chemical species was also observed during operation of the gas plasma instrument. We estimated nitrate levels to be 45.8±15.7 mg/L at 5 min and 68.0±22.7 mg/L at 15 min, and nitrite levels to be 5.45±0.76 mg/L at 5 min and 5.53±0.72 mg/L at 15 min. Our results indicated that nitrogen gas plasma generation at 1.5 kpps produced reactive chemical species as well as other factors that may be responsible for viral inactivation, including heat (45°C at 5 min and 51°C at 15 min) and UV-A emission (33.9 mJ/cm^2^ at 10 min but below the detectable limit at 5 min).

Next, adenovirus samples were treated with each of these potential inactivation factors (oxidative stress, heat, or UV radiation) individually in order to assess their respective contribution to reducing viral infectivity. Heat treatment for 5 min significantly decreased infectivity of adenovirus from 6.3 x 10^5^ PFU/ml at 35°C (Control) to 2.0 x 10^5^ PFU/ml at 50°C (S1 Fig). Moreover, samples heat treated at 70°C, used as a positive control, showed a titer of 2.0 x 10^2^ PFU/ml. This value is close to the detectable limit for the assay. Exposure of the samples to UV-A (44.79 mJ/cm^2^) at a level slightly above that seen during operation of the BLP-TES device for 5 min only partially inactivated adenovirus (i.e., viral titer of 1.4 x 10^5^ PFU/ml and 2.0 x 10^4^ PFU/ml before and after UV-A treatment, respectively). It should be noted that adenovirus was dried onto a coverslip for the UV treatment experiments, including a negative control (Control), whereas adenovirus in solution was subjected to treatment with heat or hydrogen peroxide including Control (35°C) or 0% hydrogen peroxide, respectively. Therefore, the viral titer for the Control in the UV treatment experiment corresponds to a 1 log_10_ reduction compared to those of the negative control in the experiments involving heat or hydrogen peroxide treatment. Moreover, increasing the intensity of UV-A to 236.71 mJ/cm^2^ also failed to completely inactivate adenovirus (results not shown). However, UV-C applied as a positive control reduced the infectivity of the adenovirus to a level below the detection limit of the assay (S2 Fig). Exposure to a hydrogen peroxide solution of various strength for 5 min greatly decreased infectivity of adenovirus from 1.4 x 10^6^ PFU/ml to 6.3 x 10^5^ PFU/ml at 0.0003%, 6.3 x 10^5^ PFU/ml at 0.003%, 4.3 x 10^5^ PFU/ml at 0.03%, 3.0 x 10^5^ PFU/ml at 0.3%, and 4.4 x 10^4^ PFU/ml at 3% (S3 Fig).

## Discussion

Recently, extensive research has been carried out to assess the potential application of gas plasma, derived from either helium, argon or air, for the inactivation of a broad range of pathogens including bacteria and viruses [17–23]. Gas plasma is particularly suitable for disinfection in the hospital environment, especially with respect to dangerous non-enveloped viruses, such as adenoviruses, and bacterial strains recalcitrant to standard antibiotic treatment, such as methicillin-resistant *Staphylococcus aureus* (MRSA). However, not many studies have been carried out to investigate the effect of gas plasma derived from nitrogen on microorganisms. We have previously shown that nitrogen gas plasma generated by an SI thyristor efficiently inactivates vegetative bacteria [8, 24–28] and bacterial spores [3, 29] as well as enveloped viruses [7, 30]. However, the effect of nitrogen gas plasma on non-enveloped viruses remains unclear.

In the present study, we show that adenovirus, a non-enveloped virus, is inactivated by a relatively short exposure to nitrogen gas plasma. During operation of the nitrogen gas plasma device, several factors are generated that could potentially be responsible for the observed viral inactivation including ultraviolet (UV-A) radiation, heat and the production of various reactive chemical species. Our results indicate that oxidative stress induced by the presence of hydrogen peroxide appears to be the principle mechanism of adenovirus inactivation. However, additional factors may be involved in the virucidal action of the nitrogen gas plasma. Such factors include nitrogen gas related reactive chemical species such as nitrate and nitrite, which may contribute to viral inactivation. In addition, intermediate molecular species generated during the formation of hydrogen peroxide, nitrate and nitrite may also contribute to the virucidal effect. These reactive intermediates may include NO (‧NO radicals, NO^-^, NO^+^) and its adducts (NO_2_, NO_2_^-^, NO_3_^-^, N_2_O_3_, N_2_O_4_, ONOO^-^) as well as reactive oxygen species (ROS) (e.g., O_2_^-^, H_2_O_2_) [31]. Intriguingly, NO and ROS are produced by immune systems for anti-microbial, immune modulation, cytotoxic, and cytoprotective roles [32]. Our findings suggest that nitrogen gas plasma also elicits these effects.

Previous studies showed that the main inactivation factors for cells exposed to gas plasma were UV radiation, reactive chemical products, and localized heating depending on the process gas used [7, 8, 22, 23, 26, 30, 33]. In the case of plasma derived from air, adenovirus seemed to be inactivated by reactive chemical products such as ROS and reactive nitrogen species (RNS) during the generation of gas plasma [34]. The present study using gas plasma derived from nitrogen also highlights the importance of reactive chemical products for adenovirus inactivation. These findings imply that the antiviral mechanisms of action of plasma derived from nitrogen and air are similar, perhaps because air contains almost 80% (v/v) nitrogen.

Our findings demonstrate that exposure to UV-A or heat at levels found during nitrogen gas plasma treatment for 5 min (i.e., UV-A below 33.9 mJ/cm^2^; temperature of approximately 45°C) were insufficient to inactivate adenovirus when individually applied. These results are, nonetheless, consistent with previous studies regarding the sensitivity of the virus to UV radiation. Walker and Ko reported that exposure to UV-C at an energy of 2.6 mJ/cm^2^ alone inactivated adenovirus type 2 by 60–70% [35]. For 4 log inactivation, Nwachuku et al. [36] estimated an energy of 137.9–217.1 mJ/cm^2^ was required, depending on the particular serotype under investigation. Crucially, however, nitrogen gas plasma generates UV-A but not UV-C. Our findings suggest that UV exposure resulting from nitrogen gas plasma treatment is insufficient to inactivate adenovirus.

Our results show that hydrogen peroxide at a low concentration (0.3%) efficiently inactivated adenovirus with nearly one log reduction of viral titer. Moreover, this concentration of hydrogen peroxide is similar to the levels produced during operation of the BLP-TES device. Indeed, previous studies have suggested that both the vapour and liquid forms of hydrogen peroxide at low levels effectively inactivate adenovirus [37–40]. Taken together, these findings suggest that reactive chemical products, including hydrogen peroxide, contribute to the observed virucidal effect. In addition, biochemical evaluation of nitrogen gas plasma treated adenovirus samples support this conclusion.

Biochemical analyses demonstrated that adenovirus exposed to nitrogen gas plasma undergo alterations to their viral DNA. These alterations might have arisen due to several potential factors; (i) production of reactive chemical products (ii) exposure to UV-A or (iii) elevated temperatures. However, under the experimental conditions used in the present study, elevated temperature does not contribute to adenovirus inactivation. Specifically, exposure of adenovirus to temperatures of about 45°C for 5 min had little effect on the virus, whereas adenovirus was significantly inactivated by exposure to nitrogen gas plasma for the same length of time (i.e., almost 4 log reduction of viral titer). Indeed, our findings are consistent with a previous report, which indicated that adenovirus type 5 loses infectivity only upon heating above 50°C [41]. No alterations in the capsid proteins, hexon and penton, after nitrogen gas plasma treatment were detected by immunochromatography and Western blotting.

This study suggests that the virucidal effect of gas plasma treatment may be enhanced by increasing the generation of reactive chemical products. For example, conditions used for plasma generation may be optimized to improve the inactivation of viruses by mixing gases, including oxygen, and possibly increasing the temperature. In fact, the addition of oxygen to helium has been reported to enhance the efficiency of bacterial inactivation [42]. The mechanisms involved in the virucidal effect on adenovirus may be similar to those responsible for the inactivation of bacteria. Specifically, the suggested mechanism of inactivation of bacteria by gas plasma is thought to be mainly due to the chemical interaction of NO and ROS/RNS with DNA. It should be noted that NO is known to induce DNA mutation and oxidation as well as inhibiting DNA repair and synthesis [43]. Such DNA damage will interfere with both viral gene expression and viral DNA replication.

Gas plasma is particularly suited to surface disinfection of medical devices because the plasma only penetrates to a depth of about 100–1000 nm, thereby preventing any damage to the structural integrity of the material [5]. Thus, gas plasma is a promising tool for maintaining hygiene standards in the hospital environment. However, to date, only a limited number of surface materials found in medical devices have been analyzed to study the effect of gas plasma. Further examination of the interaction between gas plasma and material surfaces will be necessary prior to the practical application of this methodology. In addition, in the present study, all inactivation experiments were performed using adenovirus-containing medium spotted onto glass coverslips. As the interaction between the viral capsid and the material surface may influence viral particle stability, further studies are required using adenoviruses spotted onto other materials to confirm the efficacy of the plasma treatment. Furthermore, the characteristics and inactivation efficiency of gas plasma can be affected by a range of different factors, including the electrical power applied, the process gas used, and the gas pressure [23]. Further optimization and scale-up may be necessary for the practical use of this technique.

According to the U.S. Environmental Protection Agency (USEPA) “Guide Standard and Protocol for Testing Microbiological Water Purifiers”, the minimum performance standards of inactivation efficiency are a 6-log reduction/inactivation of bacteria, 4-log reduction/inactivation of viruses, and 3-log reduction/inactivation of protozoan cysts [44]. The present study suggests that the nitrogen gas plasma generated by a high-voltage pulse using a SI thyristor power supply can efficiently inactivate adenovirus. Plasma treatment time of 0.940 min achieves 1 log reduction of viral titer, implying that 3.760 min is needed for up to 4 log reduction, which is an index of sufficient inactivation for viruses in the accordance with the above performance standard. Thus, nitrogen gas plasma treatment is a potentially useful method for the rapid inactivation of adenovirus. The present study suggests that the reactive chemical products produced in the gas plasma may induce damage to the viral DNA and lead to inactivation of adenovirus. Optimization of the gas plasma system to generate increased levels of reactive chemical products is expected to improve the the virucidal efficiency of this procedure.

## Supporting Information

S1 Fig Temperature dependent inactivation of adenovirus. After heat treatment (35–50°C) for 5 min, cell culture medium of adenovirus vector (AxCAwt2)-infected HEK293 cells (1.0 x 10^9^ PFU/ml) was incubated in HEK293 cells for 5 days to determine the viral titer per ml (PFU/ml) as described in Materials and Methods. Heat treatment at temperatures ≤50°C resulted in only slight inactivation of adenovirus i.e., reduction of viral titer of less than 1 log_10_. However, the temperature of samples after subjection to nitrogen gas plasma treatment for 5 min was 45°C. It should be noted that the viral titer of adenovirus decreased by more than 2 log_10_ following nitrogen gas plasma treatment for 5 min. As a negative control, adenovirus was incubated at 35°C (Control), while adenovirus incubated at 70°C (open bar) was included as a positive control.
(TIF)

S2 Fig UV-A treatment of adenovirus caused only a slight decrease in viral titer. Cell culture medium of adenovirus vector (AxCAwt2)-infected HEK293 cells (1.0 x 10^9^ PFU/ml) was spotted onto a coverslip and exposed to UV-A for 5 min using a UVGL-58 device (UVP, Upland, CA). UV-treated and untreated (Control) adenovirus samples were then used to infect HEK293 cells. Samples were incubated for 5 days to determine the viral titer per ml (PFU/ml). The results show that UV-A treatment for 5 min caused only a slight reduction in viral titer (i.e., within 1 log_10_), whereas UV-C treatment for 5 min (positive control) decreased viral titer to a level below the detectable limit for this assay.
(TIFF)

S3 Fig Inactivation of adenovirus by hydrogen peroxide (H_2_O_2_). After H_2_O_2_ treatment at the indicated concentration for 5 min, cell culture medium of adenovirus vector (AxCAwt2)-infected HEK293 cells (1.0 x 10^9^ PFU/ml) was used to infect HEK293 cells. Samples were incubated for 5 days to determine the viral titer per ml (PFU/ml). A dose-dependent decrease of viral titer was observed after treatment with 0.0003% to 0.03% H_2_O_2_. In addition, the viral titer effectively decreased by more than 1 log_10_ after treatment with 3% H_2_O_2_.
(TIFF)

S1 File Supporting Information. (DOC)
