# Supplementary figures and images for: Nitrogen Gas Plasma Generated by a Static Induction Thyristor as a Pulsed Power Supply Inactivates Adenovirus
Source: PLoS One. 2016 Jun 20;11(6):e0157922. doi: 10.1371/journal.pone.0157922 (PMC4913946; doi:10.1371/journal.pone.0157922)

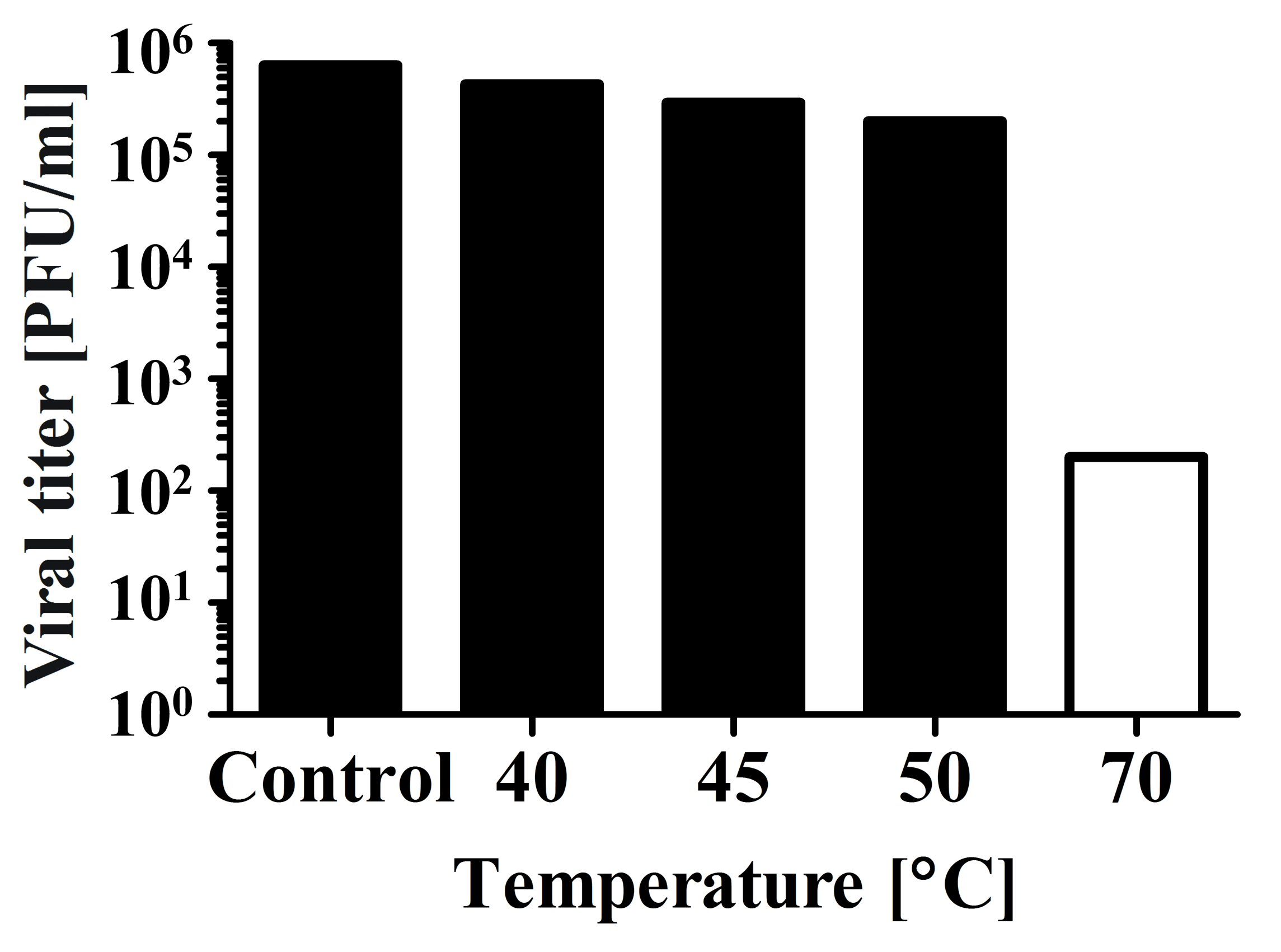

Supplement: S1 Fig — After heat treatment (35–50°C) for 5 min, cell culture medium of adenovirus vector (AxCAwt2)-infected HEK293 cells (1.0 x 10^9^ PFU/ml) was incubated in HEK293 cells for 5 days to determine the viral titer per ml (PFU/ml) as described in Materials and Methods. Heat treatment at temperatures ≤50°C resulted in only slight inactivation of adenovirus i.e., reduction of viral titer of less than 1 log10. However, the temperature of samples after subjection to nitrogen gas plasma treatment for 5 min was 45°C. It should be noted that the viral titer of adenovirus decreased by more than 2 log10 following nitrogen gas plasma treatment for 5 min. As a negative control, adenovirus was incubated at 35°C (Control), while adenovirus incubated at 70°C (open bar) was included as a positive control. (TIF) [file pone.0157922.s001.tif]

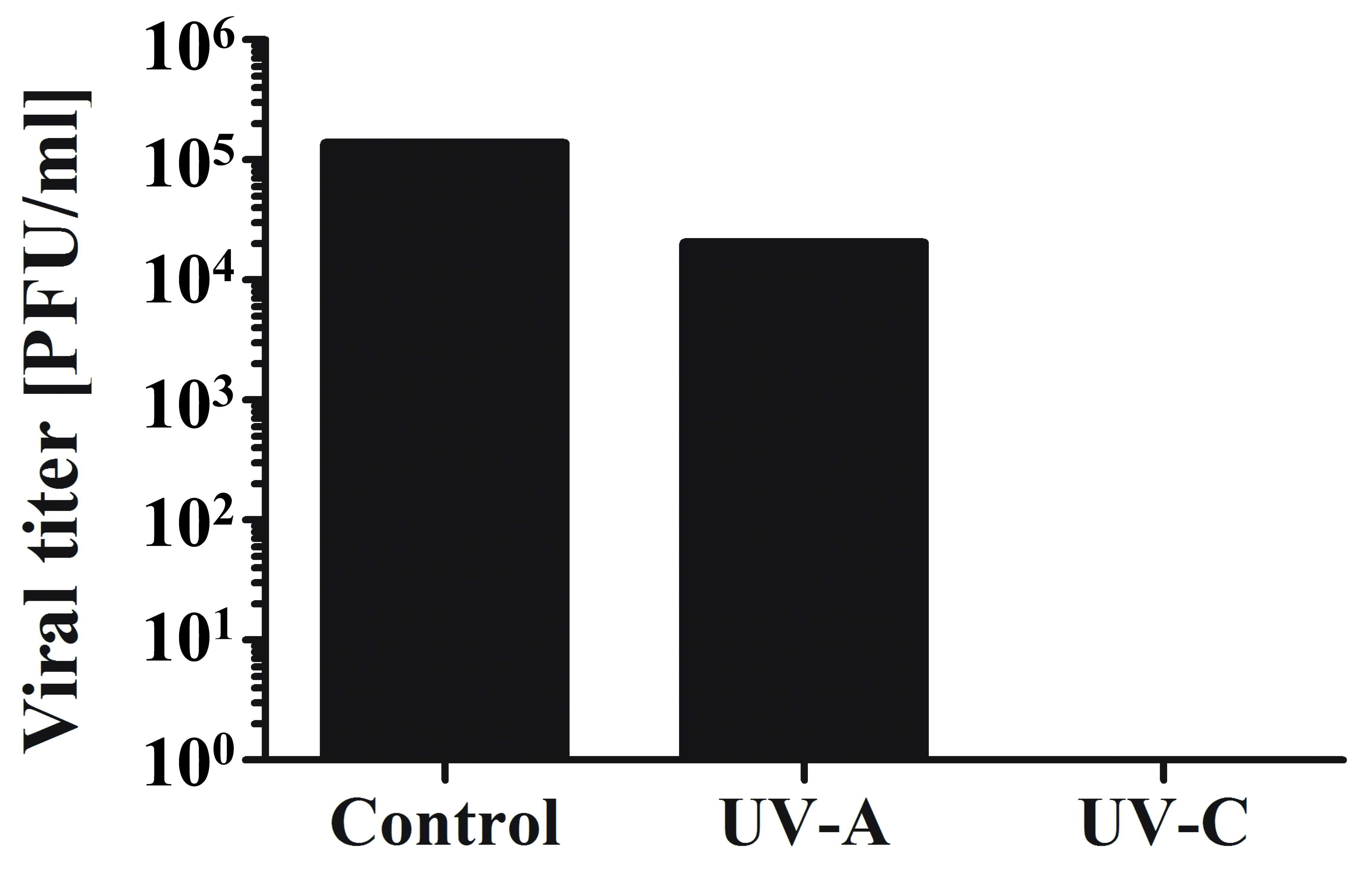

Supplement: S2 Fig — Cell culture medium of adenovirus vector (AxCAwt2)-infected HEK293 cells (1.0 x 10^9^ PFU/ml) was spotted onto a coverslip and exposed to UV-A for 5 min using a UVGL-58 device (UVP, Upland, CA). UV-treated and untreated (Control) adenovirus samples were then used to infect HEK293 cells. Samples were incubated for 5 days to determine the viral titer per ml (PFU/ml). The results show that UV-A treatment for 5 min caused only a slight reduction in viral titer (i.e., within 1 log10), whereas UV-C treatment for 5 min (positive control) decreased viral titer to a level below the detectable limit for this assay. (TIFF) [file pone.0157922.s002.tiff]

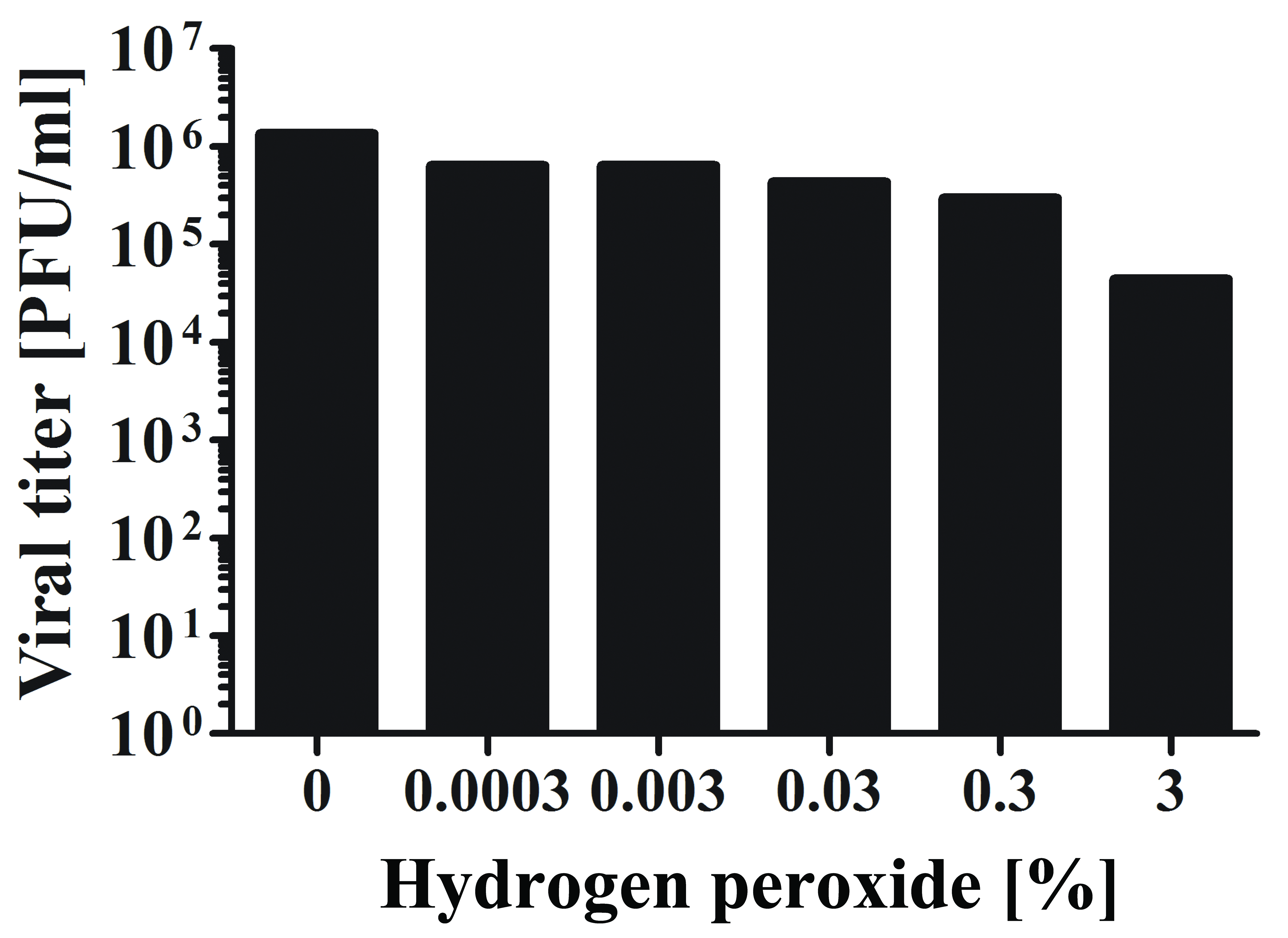

Supplement: S3 Fig — After H2O2 treatment at the indicated concentration for 5 min, cell culture medium of adenovirus vector (AxCAwt2)-infected HEK293 cells (1.0 x 10^9^ PFU/ml) was used to infect HEK293 cells. Samples were incubated for 5 days to determine the viral titer per ml (PFU/ml). A dose-dependent decrease of viral titer was observed after treatment with 0.0003% to 0.03% H2O2. In addition, the viral titer effectively decreased by more than 1 log10 after treatment with 3% H2O2. (TIFF) [file pone.0157922.s003.tiff]
