# Supplementary material for: Nitrogen Gas Plasma Generated by a Static Induction Thyristor as a Pulsed Power Supply Inactivates Adenovirus
Source: PLoS One. 2016 Jun 20;11(6):e0157922. doi: 10.1371/journal.pone.0157922 (PMC4913946; doi:10.1371/journal.pone.0157922)
Supplement: S1 File — (DOC) [file pone.0157922.s004.doc]

**S1 file**

**Supplemental Methods**

**UV-A and UV-C irradiation treatment of adenovirus**

Aliquots (20 μl) of cell culture medium containing adenovirus vector (AxCAwt2)-infected HEK293 cells (1.0 x 10^9^ PFU/ml) were spotted on glass coverslips and dried on a hot plate (HP-4530; AS-ONE, Osaka, Japan) at 35°C. The dried samples were then subjected to UV-A or UV-C treatment using a UV transilluminator UVGL-58 (UVP; Upland, CA) for 5 min at a distance of 1.3 cm. Energy dose of UV was estimated on the basis of a colour change of UV label-H indicator (Nichiyu Giken Kogyo Co., Ltd., Tokyo, Japan). The treated samples were resuspended in 20 μl of PBS, which were then used for viral titration assays.

**Hydrogen peroxide (H_2_O_2_) and heat treatment of adenovirus**

The minimum virucidal concentration of H_2_O_2_ for adenovirus was determined as follows. Aliquots (20 μl) of cell culture medium containing adenovirus vector (AxCAwt2)-infected HEK293 cells (1.0 x 10^9^ PFU/ml) were incubated with various concentrations of H_2_O_2_ at 37^o^C for 5 min. The effect of heat treatment on the infectivity of adenovirus was also investigated. Adenovirus cultures were placed on a hot plate (HP-4530; OMRON Corp., Kyoto, Japan) at 35-50 ^o^C for 5 min prior to performing a viral titration assay.
